# Nanog Dynamics in Mouse Embryonic Stem Cells: Results from Systems Biology Approaches

**DOI:** 10.1155/2017/7160419

**Published:** 2017-06-08

**Authors:** Lucia Marucci

**Affiliations:** ^1^Department of Engineering Mathematics, University of Bristol, Bristol BS8 1UB, UK; ^2^BrisSynBio, Life Sciences Building, Tyndall Avenue, Bristol BS8 1TQ, UK

## Abstract

Mouse embryonic stem cells (mESCs), derived from the inner cell mass of the blastocyst, are pluripotent stem cells having self-renewal capability and the potential of differentiating into every cell type under the appropriate culture conditions. An increasing number of reports have been published to uncover the molecular mechanisms that orchestrate pluripotency and cell fate specification using combined computational and experimental methodologies. Here, we review recent systems biology approaches to describe the causes and functions of gene expression heterogeneity and complex temporal dynamics of pluripotency markers in mESCs under uniform culture conditions. In particular, we focus on the dynamics of Nanog, a key regulator of the core pluripotency network and of mESC fate. We summarize the strengths and limitations of different experimental and modeling approaches and discuss how various strategies could be used.

## 1. Introduction

Pluripotent stem cells are defined by their ability to undergo self-renewal and capability of differentiating into all three germ layers (mesoderm, endoderm, and ectoderm). Even though during development pluripotency is a temporary condition, ad hoc cultures can sustain indefinite self-renewal of cells isolated from the inner cell mass (embryonic stem cells, ESCs) in vitro [1]. Also, it is now well assessed that somatic cells can be reprogrammed back to the pluripotent state, obtaining the so-called induced pluripotent stem cells (iPSCs), thus reverting the physiological differentiation process [2].

Given the potential of pluripotent cells in regenerative medicine applications, in the recent years, a big research effort has been put in understanding the molecular mechanisms behind ESCs decision-making. Outcomes of this research are crucial to define optimal culture conditions to push cells into the desired pluripotent or differentiated state, to optimise somatic cell reprogramming, to better understand in vivo development, and to guide the use of reprogrammed cells for regeneration purposes.

High variability in terms of functionality, gene expression, and epigenetic signature has been highlighted as a peculiar feature of both ESCs and iPSCs [3]. Focusing on gene expression variability in undifferentiated, isogenic mouse ESCs (mESCs), a number of pluripotency-related genes have been shown to be expressed heterogeneously and to present temporal fluctuations in mESCs cultured in standard serum/leukemia inhibitory factor (LIF) medium.

Nanog, a master regulator of pluripotency and development [4–6], was the first pluripotency gene for which heterogeneity and temporal fluctuations were observed [7, 8]. This was followed by the discovery of heterogeneous expression of other pluripotency factors, such as T-box 3 [9], zinc-finger protein 42 (also known as Rex1) [10], Klf4 [10], Stella [11], Esrrb [12], and *β*-catenin [13]. Importantly, the mosaic and interconvertible distribution of pluripotency genes often correlates with different degrees of potency; at population level, mESCs are fully pluripotent, but subpopulations show a different predisposition towards differentiation.

Alternative media to serum/LIF have been proposed [14]. The so-called 3i/LIF medium is serum free and contains 3 small molecules: PD0325901 (hereafter named PD), a MEK inhibitor [15]; SU5402, a FGF receptor inhibitor [16]; and CHIR99021 (Chiron), a glycogen synthase kinase-3 (Gsk3) inhibitor [17]. Intriguingly, most pluripotency genes become overall homogeneous in mESCs cultured in the 3i/LIF medium, even in the absence of FGF inhibitor (i.e., in 2i/LIF medium), and the “ground state” of pluripotency is achieved [14, 18, 19].

Is heterogeneity of Nanog and other pluripotency factors an inherent property of mESCs, fundamental for their competence to choose different fates [20], or is it an impediment to achieve standardizes cultures [21]? How do heterogeneity and complex temporal patterns of Nanog originate, and how do different culture media regulate them?

In the recent years, both experimental and computational studies have tried to address these questions. In vitro cultures are an artificial approximation of in vivo systems and self-renewal is not part of in vivo development; still, the range of pluripotent states that exists in vitro as a response to different culture conditions [1] mimics different stages of development (preimplantation embryo and late blastocyst for 2i/LIF and serum/LIF cultures, resp. [22, 23]); thus, experimental and computational studies of gene expression patterns and dynamics in both media are relevant.

Notably, many of the metastable mESC genes are, directly or indirectly, regulated by Nanog [20]; thus, in this review, we provide an updated overview of the major recent computational attempts to explain origins and functions of Nanog dynamics in mESCs, alongside with a review of available experimental data. We show that mathematical models can both aid in elucidating the mechanisms behind complex temporal gene expression dynamics and generate testable predictions.

We start reviewing works that use small transcriptional gene regulatory networks (GRNs) to explain Nanog dynamics and their role in mESC fate decision; then, we report non-GRN approaches; finally, we discuss open questions in the field and possible future research directions.

## 2. Dynamics of mESCs Explained through Transcriptional Regulatory Networks: Multistable Models for mESC Pluripotency

Notable is the work of Peterson's group in developing computational models to understand the role of transcription factor dynamics in mESC decision-making. We firstly review two early Peterson's works which consider the mESC switch between the pluripotent and the differentiated states a bistable system; Nanog heterogeneity is not considered, but the basis for formalisms developed later by the same authors to recapitulate more complex dynamics are given.

**Figure 1:**
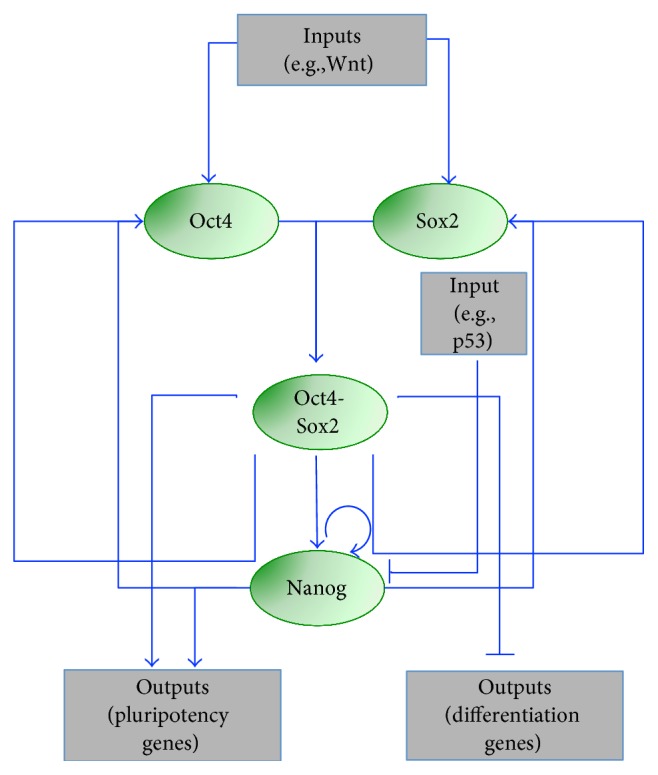
Core pluripotency regulatory network modeled in [24]. The core network includes the transcription factors Oct4, Sox2, and Nanog and the heterodimer Oct4-Sox2. Arrow- and bar-headed lines represent activation and inhibition, respectively, among genes (green ovals) and inputs/outputs (grey rectangles).

In 2006 [24], a nonlinear ordinary differential equation (ODE) model, based on the Shea-Ackers formalism [25], was developed to describe mESC decision-making in view of the dynamics of the core pluripotency network, formed by Oct4, Sox2, and Nanog [26]. The topology is composed of nested positive feedback loops, which respond to environmental signals (inputs) and act on target genes (outputs), generically indicated as stemness and differentiation genes. Oct4 and Sox2 form a heterodimer, which positively regulates both genes and Nanog [27]; also, Nanog activates itself, Oct4, and Sox2 (Figure 1()). The ODE model describing such network predicts correlated dynamics of Nanog and Oct4-Sox2. Particular input signals (such as addition of LIF or Wnt to culture) can switch both on, pushing cells to a pluripotent state, while different inputs (such as p53) can switch them off. The system shows bistability; the pluripotent and differentiated steady states are stable and mutually exclusive, meaning that the system can converge to one of the two, depending on the initial conditions and inputs. Model analysis highlights a central role of Nanog autoregulation for the core pluripotency network maintenance.

Also, the model predicts that, if Nanog is high enough, pluripotency is maintained even in the absence of Oct4 and Sox2 induction by external factors. Intriguingly, this prediction has been partly confirmed experimentally. Nanog can compensate for loss of self-renewal genes such as Esrrb, Tbx3 or Tcl1 [28], and *Oct4*^+/−^ mESCs lack Nanog-low, undifferentiated cells [29]. The model presents some limitations: it is unable to describe an effect of Oct4 overexpression in initiating differentiation [4, 30, 31], Nanog heterogeneity in the pluripotency state is not considered, and noise is neglected.

**Figure 2:**
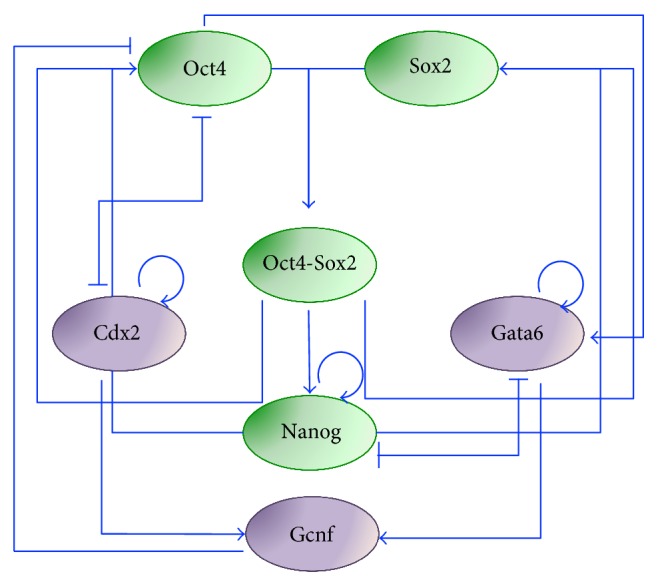
Extended regulatory network for pluripotency modeled in [32]. The network includes, in addition to the core pluripotency factors Oct4, Sox2, and Nanog, Cdx2 and Gata6 to represent trophectoderm and endoderm commitment through Oct4-Cdx2 and Nanog-Gata6 interactions, respectively, and the nuclear receptor Gcnf. Green and purple ovals represent pluripotency and differentiation genes, respectively.

In 2008, the same authors proposed an extension of the above model to better recapitulate mESC differentiation dynamics [32]. While the topology of the core pluripotency network is kept identical to the previous work, two new mutual antagonistic interactions are introduced to describe differentiation: Cdx2/Oct4 (responsible for trophectoderm lineage specification) [33] and Nanog/Gata6 (responsible for endoderm lineage specification) [34]. In addition, Cdx2 and Gata6 activate the orphan nuclear receptor germ cell nuclear receptor (Gcnf), a transcriptional repressor of pluripotency genes [35], which in turn represses Oct4. Finally, in order to account for evidence of cell tendency to specify towards an endoderm state when Oct4 is overexpressed [36], Gata6 activation by Oct4 is considered; this interaction had not been reported in the literature, so it is inserted as a model hypothesis, which is needed to recapitulate dynamics (Figure 2()). The authors developed an ODE formalism, similar to the one in their previous work [24]; the model reproduces the coexistence of three steady states, corresponding to pluripotent, trophectoderm, and endoderm cellular fates. In the absence of an external factor that forces Oct4 expression, the cells stay in the pluripotent state; the switch to a lineage-specific steady state is possible only by adding such external factor. The main achievement of this model is its ability to explain the biphasic response (“bell/inverse bell shaped”) of TFs as functions of Oct4 (i.e., they can be expressed in low/high Oct4 ranges or at intermediate Oct4 levels, only), a mechanism not easy to explain by intuition. Interestingly, Gata6 activation by Oct4 hypothesized by the authors has been later proved, experimentally, in early blastocyst development [37]. As in the 2006 model [24], Nanog is expressed homogeneously in all cells in the pluripotent state.

## 3. Experimental Evidence of Nanog Heterogeneity in Serum/LIF-Cultured mESCs

In 2005, the first experimental evidence of Nanog multimodal distribution was reported [7]. Immunofluorescence experiments showed in serum/LIF-cultured mESCs the coexistence of two subpopulations, one positive for both Oct4 and Nanog and one positive for Oct4 only. In 2007, two different studies confirmed these results and highlighted new features of Nanog heterogeneity. Singh and colleagues [38], using a cell line previously developed by Mitsui's group (*β*-geo cells, in which the beta-galactosidase-neomycin fusion gene is inserted on one allele under the endogenous Nanog promoter [6]), confirmed heterogeneity of Nanog in contrast to homogeneous Oct4 expression. Analyzing by microarray the two Nanog subpopulations, a clear pluripotency signature characterized high Nanog (henceforth called HN) cells, while low Nanog (henceforth called LN) cells showed pronounced expression of mesodermal genes. Also in 2007, Chambers and colleagues [8] generated a mESC reporter cell line for Nanog, named TNGA, obtained by inserting a GFP-IRES-Puro-pA cassette at the Nanog start codon on one allele. This study provided evidence of both multimodal Nanog distribution and its temporal fluctuations between the two states; at steady state, two distinct (GFP negative and GFP positive, corresponding to LN and HN, resp.) subpopulations appeared clearly separated and could interconvert in time.

In the following years, a number of additional reporter cell lines have been generated, and different experimental approaches, including flow cytometry and fluorescence-activated cell sorting (FACS), time-lapse microscopy, single-cell sequencing and single-molecule RNA-FISH, have been used to study Nanog distribution and dynamics at the levels of both single cells and cell populations [20].

## 4. An Excitable Model for Nanog Heterogeneity in mESCs Grown in Serum/LIF

The first mathematical model accounting for Nanog heterogeneity in mESCs grown in serum/LIF medium was presented in [39]. Kalmar and colleagues combined experiments and mathematical modeling and proposed that Nanog dynamics arise from a noise-driven excitable system.

**Figure 3:**
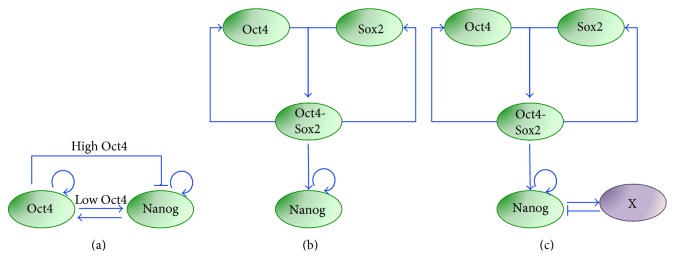
Pluripotency regulatory networks to describe excitable, bistable and oscillatory Nanog dynamics in serum/LIF. (a) Regulatory network proposed in [39] to describe Nanog dynamics in serum/LIF as excitable; additive noise is considered for Nanog and allows the system to transiently escape the HN stable steady state. (b) and (c) Networks proposed in [42] to reproduce Nanog dynamics as bistable (b) or oscillatory (c). The noise term, summed to Nanog's equation, is indispensable to reproduce Nanog heterogeneity and dynamics in (b) only. In (c), the negative feedback loop between Nanog and a not specified gene (“X”) can generate oscillatory dynamics.

Experiments were performed analyzing Nanog levels with the mentioned TNGA mESC cell line [8] both at population level by FACS and at single-cell level by time-lapse imaging. Nanog was proved to change dynamically, as sorted LN and HN mESCs were able to reproduce the original bimodal distribution, both at population level and at single-cell level; although with different timescales, sorted LN cells tended to rapidly switch to the HN state (within 24 hours), while sorted HN cells showed a slower switch off. At single-cell level, LN mESCs were able to switch Nanog on in 2-day time-lapse experiments (time lapses for the HN to LN transition were not included in the paper). Following these experimental observations, the authors derived a mathematical model showing that the HN state is stable for the system, with noise driving sporadic excursions of cells in the LN state. The system is excitable, thus highly sensible to perturbations (transcriptional noise, in this case) beyond a certain noise threshold. In detail, nonlinear ODEs describe the underlying GNR network, in which Oct4 and Nanog autoactivate their own and also each other expression and Oct4 inhibits Nanog, resulting in an overall negative feedback loop topology (Figure 3(a)). The authors assumed that Oct4 activates Nanog to saturated levels (thus this interaction is neglected in the equations) and only high levels of Oct4 can inhibit Nanog (a full biological motivation for such negative interaction, crucial for the system dynamics, is left by the authors for further investigation). The equations, based on Hill-like kinetics, consider protein dynamics only (mRNA steady-state assumption); Gaussian noise is added to the Nanog equation, in order to describe the stochastic excursions from the unique steady state of the system (HN). The model predicts that Oct4 is more homogenous in HN than in LN cells; this prediction is validated experimentally by immunofluorescence in FACS-sorted mESCs. While interesting, this result is not entirely surprising, taking into account the network topology. Finally, a stochastic model is derived and simulated using Gillespie's first reaction method. Both single-cell dynamics and population steady-state distribution of Nanog are reproduced, showing a good agreement with experimental data, and indicating that the regulation of occupancy of the two Nanog states is noise driven. The authors showed that the excitable regime is robust to parameter variations and concluded that Nanog cooperative autoregulation is crucial for the system's dynamics.

## 5. Multistable Models for Nanog Heterogeneity in mESCs Grown in Serum/LIF

In 2010, Glauche and colleagues proposed an alternative GRN-based formalism to recapitulate again experimental data obtained with the TNGA cell line. The modeled GRN includes Oct4-Sox2 heterodimer autoactivation and its activation on Nanog; also, as in Kalmar's model, Nanog autoactivation is considered (Figure 3(b)). This latter positive feedback loop, for proper parameter choices, can give raise to bistability. The stochastic differential equation (SDE) model, based on Hill kinetics, describes only protein dynamics. At steady state, the Nanog bimodal distribution observed experimentally in [8, 39] is reproduced; in time course simulations, fluctuations between the two states randomly happen due to the introduction of Gaussian noise (only in the Nanog equation). The main difference with the Kalmar model is that, in this case, both the LN and HN states are stable, with noise allowing the switch between them. Altering the noise changes the transition probability of the two Nanog states and, consequently, the residence times within each state. Interestingly, the authors simulate also the redistribution of TNGA cells upon sorting, performed experimentally in [39], matching the reestablishment of both populations on a timescale of circa 10 days. The model is finally analyzed to study the effect of Nanog heterogeneity on differentiation; the authors also consider an external differentiation signal, which inhibits Oct4 and is inhibited by Nanog, generating a double negative feedback loop. As a result of such loop, only LN mESCs respond to the differentiation signal with a decrease in Oct4-Sox2 complex level, indicating that Nanog has a “gatekeeper” role in mESC pluripotency maintenance. Of note, the differentiation signal is external; thus, no mESC spontaneous cell differentiation is conjectured. In the same paper, the authors also consider an alternative scenario in which, due to the introduction of a negative feedback loop through a not specified gene “X” that is activated by Nanog and represses it (Figure 3(c)), Nanog shows sustained oscillations, even in the absence of noise. Notably, such Nanog-mediated autorepression, hypothesized in the paper for modeling aims, has been later experimentally proven [40]. While still being able to recapitulate the experimental steady-state Nanog distribution, the oscillation scenario significantly differs in the sorting simulations: while the system is predicted to continuously shift from one steady state to the other in the fluctuation case, damped oscillations lead to the reestablishment of the steady-state distribution in the oscillation scenario. The same authors, more recently, tested experimentally these two discordant predictions, showing higher plausibility of the noise-induced bistable dynamics [41].

**Figure 4:**
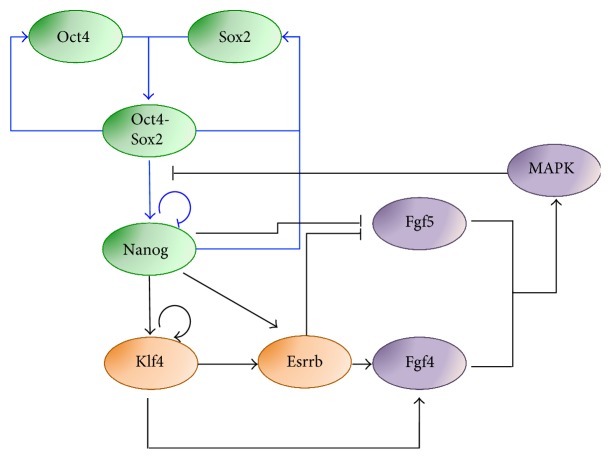
Extended regulatory network to describe Nanog multistable dynamics in serum/LIF [43]. Blue and black arrows indicate interactions of the core and extended network, respectively. The extended network interactions include additional pluripotency genes (Klf4, Esrrb), and differentiation signaling pathway (FGF and MAPK) genes.

More recently, Lakatos and colleagues proposed a different formalism to describe Nanog dynamics arising from a bistable system [43]. The authors compared dynamics of five GRN topologies compatible with experimental evidence of interactions in the core pluripotency network. In all the analyzed GRNs, the genes considered are Oct4, Sox2, and Nanog; Oct4-Sox2 is a common transcription factor for the three genes and Nanog activates Sox2 (Figure 4(), blue arrows). In addition, the following interactions are included or not: Nanog activation on Oct4 (as in earlier Chickarmane's works [24, 32]), repression of Oct4 on Nanog, and autoinhibition of Nanog (experimental evidence in [44] and [40], resp.). Of note, in all topologies, Nanog autoactivation is neglected. Quasi-steady-state approximation is used for mRNA levels, proteins are modeled using ODEs based on the Shea-Ackers representation for transcriptional regulations, and variations to parameters are introduced to account for cell-to-cell diversity. Intriguingly, modeling shows that all the topologies considered can give rise to bistability, due to the Oct4-Sox2 autoregulation and dimerization. Due to the feedback loop between Nanog and Oct4, original parameter values predict the coexistence of two steady states for both Nanog and Oct4, contradicting experimental evidence of Nanog heterogeneity in contrast to Oct4 homogeneity [45]. However, parameter manipulation can shift nullclines and reproduce the mentioned experimental data.

The authors also considered an extended topology (Figure 4, additional interactions indicated with black arrows), which includes the downstream pluripotency genes Klf4 and Esrrb (the latter acting as an amplifier [46]), fibroblast growth factor (FGF) signaling as an autocrine module, and a negative feedback through the MAP kinase/ERK kinase (MEK) cascade [47], which affects the cooperativity of the Oct4/Sox2-Nanog interaction [48–50]. Of note, FGF and MEK signaling have been proved experimentally to drive differentiation of mESCs, with a potential role in mESC heterogeneity [47]. Simulations of this extend topology show that the system allows the existence of distinct substates of Nanog and downstream transcription factors, still within the HN state; LN mESCs have higher Nanog expression than cells fully committed to differentiation, in agreement with experimental evidence [45]. Noise induces stochastic fluctuations between the multiple steady states: Nanog fluctuates in phase with Esrrb and Klf4, and in antiphase with FGF5, with no significant changes in Oct4 levels. Interestingly, multiple substates of the ON state of Nanog are predicted, suggesting the coexistence of multiple subpopulations of mESCs, responding differently to external signaling and having different pluripotency signature. The authors confirm experimentally that several components of the pluripotency network present a range of expression levels in mESC subpopulations, as also previously reported [45].

## 6. Nanog Dynamics in Serum-Free Cultures

The development of culture conditions that enable mESC ground state pluripotency (2i/LIF medium) prompted the development of new mathematical formalisms to understand the mechanisms behind the overall loss of pluripotency gene heterogeneity.

**Figure 5:**
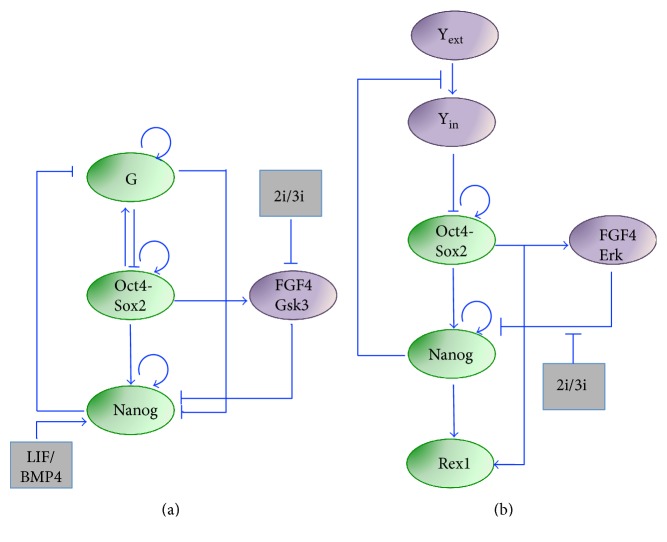
Regulatory network to recapitulate Nanog dynamics in 2i/LIF. Networks proposed and modeled in [51] (a) and [57] (b). Both systems include Nanog, the Oct4-Sox2 heterodimer, a module for FGF signaling, and the effect of the medium (2i/3i) on network regulations. In addition, in [51] (a), the interactions with LIF/BMP4 and a differentiation gene (“G”) are included, while Herberg and colleagues (b) include Rex1 as a system output only and a differentiation signal (“Y,” similar to “G” in (a)). In both models, the effects of the two inhibitors in 2i are lumped.

In 2012, the Peterson group proposed a novel model [51] to recapitulate distribution and dynamics of Nanog in different culture conditions (heterogeneity and fluctuations in LIF/bone morphogenetic protein (BMP4), homogeneity in 2i/LIF-3i/LIF) and their impact on somatic cell reprogramming (the BMP4/LIF culture condition was considered equivalent to serum/LIF [52]). In the network topology, the authors included Nanog and Oct4 autoactivations, and the activation of Oct4-Sox2 on Nanog (Figure 5(a)), as in their aforementioned previous works [24, 32]. In order to represent cells with high Oct4 levels while low Nanog [39], the previously considered Nanog activation on Oct4 and Sox2 is now removed. Also, the network includes a differentiation gene (loosely indicated as “G” in Figure 5(a)), which autoactivates itself, is activated by Oct4-Sox2 and mutually represses Nanog. Importantly, medium-regulated external factors are included: LIF/BMP4, which activates Nanog, the FGF4 receptor signaling, and Gsk3. FGF4 and Gsk3, lumped into a single variable, inhibit Nanog, are activated by the Oct4-Sox2 complex, and are blocked by 2i/3i medium (Figure 5(a)). Thus, in the overall topology, Nanog is regulated by a feed-forward loop including Oct4-Sox2 direct activation and inhibition via FGF4. An important assumption is that only when the Oct4-Sox2 heterodimer is bound to Nanog promoter additional TFs can bind (experimental evidence in [53]). A nonlinear ODEs model based on the Shea-Ackers formalism [25] is used, assuming steady-state levels for mRNA concentrations. To account for stochasticity originated within the network, the authors also formulated a master equation, simulated using the Gillespie algorithm. In BMP4/LIF medium, the system is bistable, and stochastic Nanog fluctuations arise. When Nanog levels are very low, cells are irreversibly pushed into a differentiation state, indicating again that Nanog has a pluripotency gatekeeper function. Also, the model predicts that suppression of FGF4/Gsk3 by 2i/3i can push cells into the monostable state (HN); in single-cell simulations, Nanog fluctuates around the unique steady state due to noise, but heterogeneity at population level is lost. Notably, the Nanog steady state in 2i/3i is higher than the HN steady state in BMP4/LIF medium. Of note, the noise is kept at the same level, both in BMP4/LIF and 2i/3i simulations; although LIF is not present in the 2i/3i simulations, the stem cell state is initialized with low “G,” and high Nanog and Oct4-Sox2 levels. Interestingly, the model is also used to predict somatic cell reprogramming dynamics, performing simulations upon overexpression of Oct4 and Sox2: model predictions match the experimental observation of the importance of reprogramming factor dosage for the efficiency of the process [54]. If cells are cultured in 2i, an increase in reprogramming efficiency is predicted, with synchronous Oct4 and Nanog switch-on dynamics; these predictions are in agreement with experimental evidence of an increased efficiency in the late reprogramming phase if partially reprogrammed cells are cultured in 2i/LIF medium [55, 56].

In 2014, Herberg and colleagues proposed another GRN-based modeling framework to describe medium-dependent Nanog dynamics and the resulting mESC pluripotency signature [57]. The authors extended the network topology modeled in 2010 [42] introducing (i) the pluripotency marker Rex1 [58], directly activated by both Nanog and the Oct4-Sox2 heterodimer, in order to reproduce experimental data generated using the Rex1GFPd2 cell line [59]; (ii) FGF/Erk signaling, which inhibits Nanog and is activated by Oct4; and (iii) a phenomenological external differentiation signal, which inhibits the Oct4-Sox2 complex and is inhibited by Nanog, to recapitulate differentiation dynamics (Figure 5(b)). Of note, Rex1 is only an output of the system, as it is not involved in any feedback. Overall, the topology is comparable to the one modeled by Chickarmane in 2012 [51], but here, spontaneous differentiation arising from Nanog heterogeneity is not considered: only the influence of the cellular environment is investigated.

Hill-based ODEs are derived for all genes; Gaussian noise is added to Oct4-Sox2, Nanog, and Rex1 equations; finally, steady state for mRNAs is assumed. As in the Chickarmane et al. 2012 paper, the system presents bistability, abrogated when culture medium is switched to 2i (LIF is implicitly considered here). In particular, in serum/LIF (FGF/Erk active), Nanog shows bistability due to its positive autofeedback loop, which strength is adjusted in order to have a steady-state distribution of circa 20% LN and 80% HN cells, as observed in TNGA experiments [8, 39]; the model can also reproduce Rex1 distribution measured culturing Rex1GFP2 mESCs. In time simulations, noise allows the switch between the HN and LN states, which is predicted to happen in the timescale of days. Increasing the level on noise results in more frequent switch and raised proportion of LN cells. Parameters of the Oct4-Sox2 complex are adjusted to keep its level constantly high and homogeneous.

When simulating the 2i condition, the block of Erk (due to the presence of PD in the medium) reduces the inhibition of Nanog, pushing the system outside the bistability region into the “ground pluripotency” state, characterized by high Nanog, Oct4-Sox2, and Rex1 and low FGF/Erk. Of note, the effect of Chiron in the 2i medium is not considered. The model is used to predict differentiation dynamics: after the first 24 hours of 2i withdrawal, Nanog decreases with fast dynamics, followed by Rex1 and, ultimately, Oct4-Sox2, matching experimental observations [60]. Experimental data in the paper validate model predictions about the asynchronicity of the differentiation process, possibly due to individual cell variability arising when the inhibitor is removed from the medium. A comparison with differentiation dynamics of cells cultured in serum/LIF (thus, starting from bimodal Nanog and Rex1 distribution) is not reported.

## 7. A Probabilistic Model for Nanog Multistationary Dynamics

Luo and colleagues [61] proposed an alternative approach to describe medium-depended Nanog dynamics in mESCs. Differently from the above formalisms, the authors did not rely on a regulatory network, to avoid introducing assumptions in case of lacking or contrasting data about underlying regulations, and possible overparameterization. Instead, they used a statistical model, based on probability density functions and Gaussian noise. The model has just one variable (representing Nanog), for which the existence of a stationary distribution is assumed (represented as a mixture of Gaussian distributions). Using nested sampling, model parameters are fitted on novel experimental data presented in the paper: the authors identify in TNGA mESCs the distribution of Nanog as a mixture of high, medium, and low (HN, MN, and LN, resp.) subpopulations. Then, they focus on the MN state, which is shown by experiments to have the highest dynamical changes. Nanog dynamics are measured in sorted MN cells replated in different culture conditions for four days (LIF/BMP4, PD only, Chiron only, and 2i/LIF); Nanog distribution shifts towards the HN state in the 2i/LIF condition and these data are used to validate model predictions about sorting dynamics. Intriguingly, Chiron widens the LN and MN states, while PD narrows them. One interesting result of the fitting is a competitive effect of PD and Chiron: while the first tends to add noise to the system, the second can filter noise. The approach used in this paper is definitively interesting: it allows inferring directly the shape of the potential function from the data, without a priori knowledge of the underlying signaling network. The experiments were performed 4 days after the sorting; it could be of interest to perform both experiments and simulations on a longer timescale, to check if the distributions reported in the paper are or not at steady state.

## 8. Nanog Heterogeneity Arising from Allelic Switching

Miyanari and Torres-Padilla [62] recently suggested an alternative mechanism contributing to Nanog heterogeneity based on allelic regulation. The authors firstly analyzed Nanog nascent transcription by RNA fluorescent in situ hybridization (RNA-FISH) in vivo, observing monoallelic and biallelic expression in preimplantation embryo and in late blastocyst, respectively. To study the dynamic switch of Nanog in vitro, the authors generated a novel reporter cell line based on a dual-reporter system (named NGR mESCs), in which two distinct fluorescent reporters (destabilized TurboGFP and mCherry) are inserted downstream of the NANOG-coding region in the two alleles. In NGR mESCs cultured in serum/LIF, Nanog transcriptional firing was observed, with an odd distribution of allelic firing: only a very low percentage of cells showed Nanog biallelic expression, in contrast to biallelic expression of other pluripotency genes such as Oct4. Consequently, in vitro cultures showed distinct populations for Nanog: monoallelic, biallelic, and no expression; similar results were obtained using single-molecule RNA-FISH (smRNA-FISH) [63]. In contrast, biallelic expression was reported in 2i/LIF. These results suggested a crucial role of allelic expression, and the possibility of achieving ground state pluripotency by activating the second Nanog allele.

**Figure 6:**
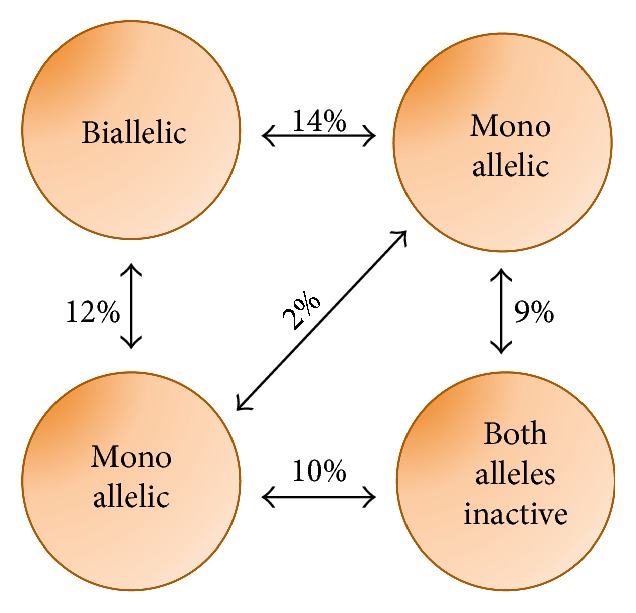
Schematic of Nanog allelic expression, as modeled in [64]. mESCs are grouped into 4 types given different patterns of Nanog allelic expression; the percentage fraction of mESCs residing in each group is indicated as well as the transition probability (in percentage).

In light of such results, Wu and Tzanakakis proposed a mathematical model to recapitulate medium-dependent Nanog dynamics [64]. The mESC population is divided into 4 groups (biallaleic, monoallelic, and both alleles inactive), with percentages of residence in each group and probabilities of transition among groups extrapolated from RNA-FISH and allele-specific RT-PCR experiments in [62] (Figure 6).

The stochastic allelic switching is modeled using a homogeneous Markov chain, assuming that the next state does solely depend on the current state; asynchronous proliferation rates are considered.

The model is simulated using a Monte Carlo algorithm, setting the half-life of endogenous Nanog to 2 hours, as measured in [65]. In the absence of noise, at population level, Nanog distribution shows 3 peaks (low for both alleles inactive, middle for monoallelic, and high for biallelic cells), while addition of noise leads to a bimodal distribution (LN and HN only). In single-cell-simulated dynamics, HN cells show a slow (circa 20 hours) switch off, while LN cells switch on in circa 5 hours. The predicted dynamics are much faster than the ones previously observed with TNGA mESCs; thus, the authors simulate the expected output of the latter cells, in which the reporter is inserted only in one allele and has half-life longer than endogenous Nanog. In this case, even in the absence of transcriptional noise, only 2 peaks in the reporter distribution are predicted, confirming experimental observations in [8, 39]. Also, the predicted correlation between endogenous Nanog and the reporter dynamics is very high (Pearson coefficient = 1) if both alleles are tagged and the half-life of the reporter is comparable to the one of Nanog, while correlation is lost (Pearson coefficient = 0.06) using a TNGA-like tagging approach. Finally, the authors use the model to simulate dynamics of Nanog^+/−^ cells, predicting that Nanog deletion on one allele can impact its distribution at population level. The paper does not include results about Nanog dynamics in 2i/LIF, but the authors mention that the model could easily be extended to describe the latter case by properly changing the proportion of cells in each subpopulation. Simulation results about the impact of differences between endogenous and reporter kinetics could explain mismatches in steady-state distributions and dynamics observed using different Nanog-tagged cell lines. Such predictions have been partly corroborated recently: the group of Henrique generated a novel reporter cell line (Nd mESCs), which contains a transgenic bacterial artificial chromosome (BAC) with a destabilized Venus reporter protein (Venus-Nuclear-PEST) under the control of Nanog regulatory regions [66]. Nd mESCs differ from TNGA cells for two main reasons: the two endogenous Nanog alleles are kept intact and the half-lives of the reporter mRNA and protein are comparable to the endogenous Nanog ones (circa 4 and 2 hours, resp.). In Nd mESCs grown in serum/LIF, as compared to TNGA cells, Nanog expression is again mosaic but within an overall narrower distribution; also, fluctuations between the LN and the HN state and restoration of the original distribution from LN- and HN-sorted cells occur in a shorter timescale (circa 4 hours and 2–4 days of culture, resp. [66]).

## 9. Nanog Dynamics Arising from a Protein Interaction Network

**Figure 7:**
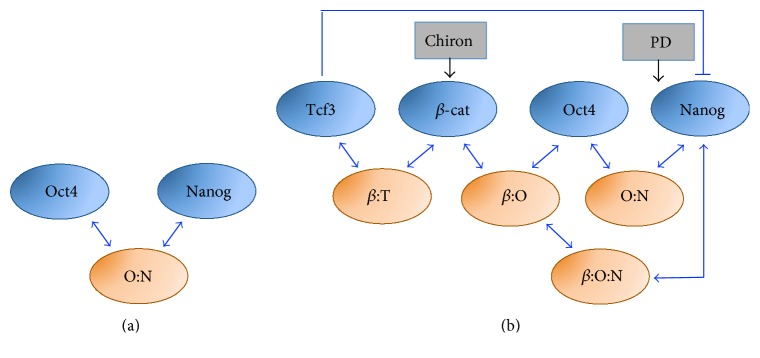
Pluripotency protein interaction networks, as modeled in [67]. Scheme of the minimal (a) and refined (b) protein interaction networks. O:N, *β*:T, *β*:O, and *β*:O:N indicate Oct4-Nanog, *β*-catenin-Tcf3, *β*-catenin-Oct4, and *β*-catenin-Oct4-Nanog complexes, respectively.

Recently, Muñoz-Descalzo and colleagues [67] considered an alternative model to describe Nanog dynamics in mESCs focusing on posttranscriptional interactions. In view of recent data generated by the same research group about the key role of protein balance for mESC pluripotency maintenance [68], the authors reanalyzed the excitable model of Kalmar and colleagues and pointed out its failure in reproducing (i) the increased correlation between Oct4 and Nanog observed by single-cell immunofluorescences in 2i/LIF medium and (ii) a critical region characterized by low levels of both Nanog and Oct4, in which pluripotency is lost. The authors started considering a minimal model, named NOC (Figure 7(a)), which encompasses only Oct4 and Nanog, and assume that the correlation between the two proteins results from the formation of a protein complex (O:N) previously described experimentally [69–71]. In the model, Oct4 and Nanog proteins exist either free or bound together in a complex; transcriptional regulations are neglected. Importantly, the model takes into account different stabilities for proteins (high for Oct4 and Nanog/Oct4 complex and low for Nanog), measured experimentally in the paper. To account for transcriptional heterogeneity of Nanog, its transcriptional bursting [40, 62] is considered. The resulting discrete stochastic formalism is simulated for both serum/LIF and 2i/LIF conditions by tuning Nanog expression (infrequent transcriptional bursting in serum/LIF and high frequency bursting in 2i/LIF). The model correctly matches Nanog and Oct4 correlation in the two media, and Nanog mRNA distribution (unimodal in both media, but shifted towards high levels in 2i/LIF) observed experimentally by mRNA-FISH. Still, the model has some pitfalls: it predicts that knock-out of Nanog results in increased Oct4 levels, the latter experimentally associated with differentiation, contradicting experimental evidence about mESC ability to maintain pluripotency even in absence of Nanog [8].

Thus, the authors developed a refined model (named TBON), which also includes Tcf3 and *β*-catenin, two Wnt pathway proteins associated with pluripotency [59, 72, 73] and involved in a protein-complex with Oct4 [74, 75]. The new topology (Figure 7(b)) considers, in addition to the Oct4-Nanog complex (O:N), the inducers (PD and Chiron) and three additional complexes: *β*-catenin with Oct4 (*β*:O) [74, 75], *β*-catenin with Tcf3 (*β*:T) [76], and *β*-catenin with Oct4 and Nanog (*β*:O:N).

Stochastic simulations of the model match protein distributions and correlations of protein levels in both serum/LIF and 2i/LIF. The model shows that, in 2i/LIF, ground state pluripotency is achieved by attenuation of free Oct4, thus limiting its effect in promoting differentiation. Also, the model predicts that, in absence of Nanog, the *β*:O complex becomes stronger, enabling cells to maintain pluripotency, as confirmed in experiments reported in the paper. In case of lack of *β*-catenin, the model predicts that the correlation between Nanog and Oct4 is unchanged, but their levels are lower, due to Tcf3 increase. These model predictions are in agreement with experimental evidence about the not absolute requirement of *β*-catenin for pluripotency [77], although its lack makes mESCs more prone to differentiate [59]. Conversely, abrogation of Tcf3 sustains pluripotency through increased Nanog, decreased Oct4 and *β*-catenin, and impaired Oct4-Nanog correlation. Finally, the model predicts that the removal of Oct4 from the system causes a drop in Nanog levels (as, if not bound in protein complex, Nanog has faster degradation), in agreement with loss of pluripotency in Oct4^−/−^ cells [31].

## 10. Limitations of Existing Formalisms for Ground State Pluripotency Cultures

All the reviewed models but the Muñoz-Descalzo et al. one just consider the molecular effect of the MEK inhibitor (PD), neglecting effects of the Gsk3 inhibitor (Chiron) in ground state pluripotency cultures. Given the failure of PD alone in sustaining mESC clonal propagation [1, 59] and the crucial functions of the Wnt/*β*-catenin pathway in pluripotency maintenance and successful reprogramming of somatic cells to pluripotency [13, 78–82], the role of the Wnt pathway in cellular heterogeneity and mESC plasticity should be better characterized. Also, possible effects of nonhomogenous, or cell density-dependent, drug uptake in cellular cultures on the system dynamics might be considered. Finally, no current model accounts for a certain level of Nanog heterogeneity and temporal fluctuations persistent in 2i/LIF recently observed experimentally [83, 84]. It remains to be shown whether long-term cultures in 2i/LIF could impair mESC karyotypic and epigenetic stability, given prolonged exposure of cells to Gsk3 inhibition [85]. Recently, R2i, an alternative serum-free, chemical media, has been proposed to sustain mESC ground state pluripotency [86]. Of note, also R2i eliminates mESC heterogeneity, but it acts on the pluripotency circuit through other routes (it contains inhibitors of TGF*β* and FGF signaling pathways); this provides a good indication that mosaic expression of pluripotency genes in serum/LIF might originate from multiple sources, which mathematical formalisms should consider.

## 11. Crosstalk between Gene Expression Dynamics and the Cell Cycle

Mathematical models could be particular useful in elucidating the complex interconnection between cell cycle, the pluripotency network, and cellular fate. In the experimental works mentioned in this review, fluctuations of Nanog reporter genes were observed within a mESC cell cycle [62, 84]; however, there is also evidence of coupling between pluripotency network gene dynamics and the cell cycle, as recently reviewed in [87]. In an early work reporting Nanog heterogeneity [38], FACS-sorted HN cells were found to upregulate cell cycle genes characteristic of the S-G2 phases, while LN cells expressed genes characteristic of the G1 phase. Also, MacArthur and colleagues, using an inducible system, found a correlation between Nanog and cell cycle checkpoints genes [60]. Recently, Nanog but not Oct4 expression was proved to oscillate in mESCs synchronized for the cell cycle [88]. In a recent work [41], Herberg and colleagues extended their previous GRN model [57] to include mESC proliferation; the resulting agent-based model predicts an effect of different cell cycle times in the proportion of cells in low-high Nanog subpopulations in serum/LIF; analysis in ground state pluripotency cultures (in which mESC cell cycle is known to differ [89]) was not performed.

Multiscale modeling approaches, able to account simultaneously for processes at subcellular, intercellular, and population levels, could be highly informed by quantitative single-cell measurements (such as live imaging and sequencing), and be able not only to reproduce experimental data but also to generate useful predictions, usable for targeted control of mESC fate, both in pluripotency maintenance and in differentiation.

## 12. Conclusions and Future Perspectives

Here, we have reviewed recent computational/experimental results about mechanisms and consequences of Nanog dynamics in populations of isogenic mESCs. We reported main assumptions, results and predictions of mathematical models based on regulatory networks (in which Nanog dynamics result from its interactions with other pluripotency genes, signaling pathways, and drugs at transcriptional and posttranslational levels [39, 42, 43, 51, 57, 67]), statistical models [61], and unbalanced Nanog allelic expression [64]. A common conclusion is that Nanog acts as a molecular gatekeeper, fine-controlling cell fate in response to pluripotency and differentiation genes' regulations, internal noise, and external stimuli. We showed that different formalisms are able to reproduce Nanog dynamics observed experimentally with reporter mESCs cultured in both serum/LIF and chemical, serum-free culture media, and generate testable predictions.

Still, many open questions remain regarding both discordant experimental results and the validity of modeling assumptions. How can models based on different GRN topologies reproduce the same experimental data? One critical step in deriving mathematical models is parameterization. Notably, given the same system of equations, different set of parameters and timescales of the variables involved can result in completely different dynamic scenarios; conversely, different sets of genes can be included in GRNs to reproduce specific dynamics, as far as the topology encompasses key elements (e.g., positive feedback loop(s) for bistability). It is therefore crucial, as far as possible, to use parameters directly measured and rely only on interactions unambiguously identified experimentally.

Also, when comparing simulations to experimental data, it is vital to critically account for the experimental settings and the timescales of the considered variables. Among the models reviewed here, only Wu and Tzanakakis explicitly took into account the differences in the degradation rates of endogenous Nanog and its reporter; their results suggest modeling as a powerful tool for experimentalists to infer endogenous dynamics from fluorescent reporter data. The Wu and Tzanakakis paper, as the Luo and colleagues one, is for sure interesting also for being able to describe Nanog dynamics at population level starting from single-cell dynamics, without relying on a GRN model with consequent assumptions on the network topology. However, the Luo et al. model, while capable of reproducing Nanog heterogeneity, cannot explain its source.

Focusing on timescales, the commonly used steady-state assumption for mRNA dynamics, while reducing the parameter space, might lead to misrepresentative results. Indeed, in 2014, two independent reports [90, 91] focused on Nanog distribution at the protein level and found that mESCs cultured in serum/LIF do not show protein biallelic expression, in contrast with mRNA firing measured in [62]. In particular, in [91], Filipczyk and colleagues generated mESCs encoding dual fusion proteins to the Nanog C-terminus, with green and red fluorescent reporters placed on the two alleles. At cell population level, FACS analysis showed bimodal distribution for Nanog in mESCs cultured in serum/LIF, although with a narrower expression range as compared to TNGA cells (as with Nd cells), but correlated expression of the two fusion proteins, suggesting biallelic expression of Nanog protein. These results are in agreement with examination of mature cytoplasmic RNA transcript, which was found to be biallelic regardless allelic Nanog firing [63]. Given that the fusion fluorescent proteins generated by Filipczyk and colleagues have an half-life comparable to the endogenus Nanog's one, protein biallelic expression can be compatible with the mRNA allelic switching reported in [62] only if different timescales for mRNA and protein are considered [20]. Taken together, these results suggest that the quasi-steady-state assumption for Nanog mRNA dynamics might be revised and that GNR feedback mechanisms are still needed to explain heterogeneous Nanog protein dynamics in serum/LIF.

Also, in our view, the impact of intrinsic and extrinsic noise on mESC fate should be better addressed: molecular instability is often represented incorporating a stochastic term in a set of differential equations, but whether it is a good representation has not been addressed. It would be extremely interesting to combine quantitative estimate of noise (as in [92]) with GRN-based formalisms.

Scaling up the reviewed mathematical models for small GRNs, involving other key genes involved in pluripotency and differentiation identified with bioinformatics and statistical methods [60, 93–95] and unraveling their role in mESC temporal dynamics, remains an open challenge. Also, the descriptive and predictive power of mathematical models could be significantly increased accounting also for the combined role of noncoding RNAs [96–99], epigenetic mechanisms [100], metabolism [101], and posttranscriptional modifications [102].

From a more broad prospective, we do believe that the questions about the role of heterogeneity in mESC decision-making stated at the beginning of this review have not yet been fully addressed. Xenopoulos et al. [103], using high-resolution live cell imaging, reported “salt and pepper” Nanog pattern in early blastocyst, but lack of temporal fluctuations, and irreversible commitment to epiblast and primitive endoderm. If and how heterogeneity can be advantageous for mESC plasticity is currently not known; the principles behind the use of noise and fluctuations by genetic circuits, also in an evolutionary prospective, are beginning to be elucidated only recently [104]. The hypothesis that pluripotency is an emergent property of a population of cells rather than a characteristic of a single cell [60], with noise and cellular heterogeneity conferring stem cell high entropy, and thus the potential to choose a number of specialized, differentiated fates [105, 106], still needs to be more extensively characterized not only for embryonic but also induced and adult stem cells. Also, the “exploratory hypothesis” for pluripotent cells, which conjectures stem cell decision-making as a two-step process in which firstly stochasticity induces a critical state that primes diverse transcriptional programs, and then one particular fate is chosen via interaction with external inputs [107, 108], represents, in our view, an interesting research avenue for further investigation.
